# Attitude-Orbit Coupled Control of Gravitational Wave Detection Spacecraft with Communication Delays

**DOI:** 10.3390/s23063233

**Published:** 2023-03-17

**Authors:** Yu Zhang, Yuan Liu, Jikun Yang, Zhenkun Lu, Juzheng Zhang

**Affiliations:** 1 MOE Key Laboratory of TianQin Mission, TianQin Research Center for Gravitational Physics & School of Physics and Astronomy, Frontiers Science Center for TianQin, Gravitational Wave Research Center of CNSA, Sun Yat-sen University (Zhuhai Campus), Zhuhai 519082, China; 2 School of Aeronautics and Astronautics, Sun Yat-sen University, Shenzhen 518107, China

**Keywords:** attitude-orbit coupled control, spacecraft formation, distributed coordination controller, dual quaternion

## Abstract

In order to meet the position and attitude requirements of spacecrafts and test masses for gravitational-wave detection missions, the attitude-orbit coordination control of multiple spacecrafts and test masses is studied. A distributed coordination control law for spacecraft formation based on dual quaternion is proposed. By describing the relationship between spacecrafts and test masses in the desired states, the coordination control problem is converted into a consistent-tracking control problem in which each spacecraft or test mass tracks its desired states. An accurate attitude-orbit relative dynamics model of the spacecraft and the test masses is proposed based on dual quaternions. A cooperative feedback control law based on a consistency algorithm is designed to achieve the consistent attitude tracking of multiple rigid bodies (spacecraft and test mass) and maintain the specific formation configuration. Moreover, the communication delays of the system are taken into account. The distributed coordination control law ensures almost global asymptotic convergence of the relative position and attitude error in the presence of communication delays. The simulation results demonstrate the effectiveness of the proposed control method, which meets the formation-configuration requirements for gravitational-wave detection missions.

## 1. Introduction

In recent years, space gravitational-wave detection has become an important research focus to confirm general relativity and open a window to gravitational-wave astronomy. Currently, the most popular space gravitational-wave detection missions include the LISA program [1,2] in cooperation with Europe and the United States, the DECIGO program [3] in Japan, and the Tianqin [4] and Taiji programs [5] in China.

In general, these missions consist of three spacecrafts. They form the shape of an equilateral triangle, with two test masses inside each spacecraft as the endpoints of the Michelson interferometer. In order to detect gravitational waves, the distance change between test masses within different spacecrafts is required to be as small as possible. However, there are some situations where spacecraft motion surpasses scientific-mission requirements. For example, when the spacecraft has an entry error and the test mass has a release error, or the spacecraft deviates from the desired states due to external disturbance force. Consequently, the position and attitude of the spacecraft and the test masses need to be controlled to meet the requirements of scientific measurements before starting a scientific mission. High-precision satellite orbit determination is one of the necessary conditions to achieve high-precision control. Some space missions, such as GRACE [6] and BepiColombo [7], use accelerometers to perform a pseudo-drag-free spacecraft orbit determination, which provides an important reference value for high-precision control of gravitational-wave detection. The spacecrafts and test masses are considered rigid bodies, and their control actuators are microthrusters and electrostatic actuators, respectively. It is known that coupling exists between the rotation and translation [8]. In order to achieve high control accuracy in the system, the translation and the rotation of the spacecraft and test masses should be, simultaneously, taken into account. Moreover, the long distance between the spacecrafts makes it necessary to consider communication delays between them. Therefore, the attitude-orbit coordination control of multiple rigid bodies with communication delays is investigated in this paper.

As a distributed cooperative control strategy, consensus algorithms have recently been studied extensively in the cooperative control of multi-spacecraft systems. The basic idea for information consensus is that members in the system obtain information from other neighbors and generate control strategies based on their status, to ensure the consistency of specified status in the entire system. In Ref. [9], a distributed attitude coordination controller based on a second-order consistency algorithm was designed under the directed communication topology. Based on an extended state observer, Yang et al. [10] developed a nonlinear attitude tracking control approach to achieve attitude consensus control. Min et al. [11] studied adaptive attitude synchronization of spacecraft formation with communication delays. However, attitude control is only considered in the literature mentioned above. To improve control accuracy in formation systems, it is necessary to consider the coupled effects between attitude and orbit motions.

It is well-known that coupled attitude-orbit modeling is one of the core technologies for distributed coordination control. Numerous research results have been published regarding the modeling of spacecraft formation [12,13,14,15,16]. In the previous literature, the modeling of orbit and attitude are considered separately. However, it is important to consider the strong coupling characteristics between the orbit control and the attitude control. In recent years, the special Euclidean group SE(3) [17,18,19] and dual quaternions [20,21,22] have been the most popular methods to describe the coupling motion of rigid bodies. A 4 × 4 homogeneous transformation matrix is utilized when modeling rigid bodies on SE(3), while the model is described more compactly by dual quaternions, which have only 8 parameters, and the dual-quaternions multiplications have a lower computational cost than homogeneous transformation matrix multiplications [23]. Wang et al. [24] proposed a quaternion solution for attitude and position control of rigid-bodies’ networks, which was the first attempt to apply the dual-quaternion representation to the study of formation-control problems. In Ref. [25], the leaderless-consistency and static-leader-consistency problems were investigated using dual quaternions for networked fully actuated rigid bodies. On this basis, a distributed control law was proposed with a time-varying leader [26]. In the field of robotics, Savino et al. [27] proposed a solution to the pose-consistency problem of multi-rigid-body systems based on dual quaternions.

Nevertheless, to the best of our knowledge, the work mentioned above rarely considered the communication-delays problem under the attitude-orbit coupled control of multiple-spacecraft formation. In the gravitational-wave detection mission, the distance between spacecrafts is more than one hundred thousand kilometers, and the communication delay will seriously reduce the real-time performance of the controller, thereby reducing control accuracy. The coupling effect between orbital motion and attitude motion is also an essential factor affecting control accuracy. Therefore, it is necessary to design a coordination controller for the spacecrafts and the test masses, considering both the attitude-orbit coupling effect and the communication delays between the spacecrafts. In this paper, the dual quaternion is used as the primary mathematical tool to establish the attitude and orbit coupling dynamic model of the spacecrafts and the test masses. Then, the full-state feedback control strategy is used to track the desired position and attitude of the spacecrafts and the test masses, and the consensus algorithm is used to achieve the coordinated control between them. The communication delays between spacecrafts are considered in the cooperative control process. The main contribution of this paper is the proposal of a distributed coordinated control law by combining the full-state feedback control strategy with the consensus algorithm while considering the communication delays.

The remainder of this paper is organized as follows. Section 2 gives material background, as well as the dynamics of the attitude-orbit coupled relative motion of the spacecrafts and test masses based on dual quaternions. The coordination controller is designed and the controller’s stability is demonstrated using the Lyapunov function in Section 3. Finally, numerical simulation results are presented in Section 4, followed by conclusions in Section 5.

## 2. Material Background and Relative Coupled Dynamics

### 2.1. Quaternions and Dual Quaternions

As an extension of complex numbers, quaternions can be defined as $q=q_{0}+q_{1}i+q_{2}j+q_{3}k$, where $q_{0}$, $q_{1}$, $q_{2}$, $q_{3}\in R$, and *i*, *j*, *k* satisfy the following properties: $i^{2}=j^{2}=k^{2}=-1$; i=jk=−kj; j=ki=−ik. Let $H=\{q:q=\left(\xi ,\bar{q}\right)\}$ denote the set of quaternions, where $\xi =q_{0}$ and $\bar{q}=\left[q_{1},q_{2},q_{3}\right]\in R^{3}$ are the scalar and vector part of the quaternions, respectively. The set of vector quaternions and scalar quaternions are defined as $H_{v}=\left\{q\in H:\xi =0\right\}$, and $H_{s}=\left\{q\in H:\bar{q}=0\right\}$, respectively. Hence, the quaternion $\left(xi+yj+zk\right)\in H_{v}$ represents the point $\left(x,y,z\right)\in R^{3}$.

A dual number is defined as $\hat{a}=a_{r}+\varepsilon a_{d}$, where $a_{r},a_{d}\in R$ are the real part and the dual part, respectively. ε represents a dual unit satisfying $\varepsilon^{2}=0$ but ε≠0.

The set of dual quaternions, dual vectors, and dual scalar quaternions are defined as $\mathrm{DQ}=\{\hat{q}:\hat{q}=q_{r}+\varepsilon q_{d},q_{r},q_{d}\in H\}$, ${\mathrm{DQ}}_{v}=\left\{\hat{q}:\hat{q}=q_{r}+\varepsilon q_{d},q_{r},q_{d}\in H_{v}\right\}$, and ${\mathrm{DQ}}_{s}=\left\{\hat{q}:\hat{q}=q_{r}+\varepsilon q_{d},q_{r},q_{d}\in H_{s}\right\}$, respectively. The set of dual scalar quaternions with zero dual part is denoted by ${\mathrm{DQ}}_{r}=\left\{\hat{q}:\hat{q}=q+\varepsilon 0,q\in H_{s}\right\}$.

Given are two quaternions, $q_{1}=\left(\xi_{1},{\bar{q}}_{1}\right)$ and $q_{2}=\left(\xi_{2},{\bar{q}}_{2}\right)$, in H, two dual quaternions ${\hat{q}}_{1}=q_{1r}+\varepsilon q_{1d}$ and ${\hat{q}}_{2}=q_{2r}+\varepsilon q_{2d}$ in DQ with $q_{1r}$, $q_{1d}$, $q_{2r}$, and $q_{2d}$ in H. The addition, multiplication, conjugation, dot product, and cross product are defined, respectively, by

(1) $$\begin{array}{ccc} q_{1}+q_{2} & = & (\xi_{1}+\xi_{2},{\bar{q}}_{1}+{\bar{q}}_{2})\in H \end{array}$$

(2) $$\begin{array}{ccc} {\hat{q}}_{1}+{\hat{q}}_{2} & = & \left(q_{1r}+q_{2r}\right)+\varepsilon \left(q_{1d}+q_{2d}\right)\in \mathrm{DQ} \end{array}$$

(3) $$\begin{array}{ccc} q_{1}⊗q_{2} & = & (\xi_{1}\xi_{2}-{\bar{q}}_{1}\cdot {\bar{q}}_{2},\xi_{1}{\bar{q}}_{2}+\xi_{2}{\bar{q}}_{1}+{\bar{q}}_{1}\times {\bar{q}}_{2})\in H \end{array}$$

(4) $$\begin{array}{ccc} {\hat{q}}_{1}⊗{\hat{q}}_{2} & = & \left(q_{1r}⊗q_{2r}\right)+\varepsilon \left(q_{1r}⊗q_{2d}+q_{1d}⊗q_{2r}\right)\in \mathrm{DQ} \end{array}$$

(5) $$\begin{array}{ccc} q^{*} & = & (\xi ,-\bar{q})\in H \end{array}$$

(6) $$\begin{array}{ccc} {\hat{q}}^{*} & = & {q_{r}}^{*}+\varepsilon {q_{d}}^{*}\in \mathrm{DQ} \end{array}$$

(7) $$\begin{array}{ccc} q_{1}\cdot q_{2} & = & \left(\xi_{1}\xi_{2}+{\bar{q}}_{1}\cdot {\bar{q}}_{2},\bar{0}\right)\in H_{s} \end{array}$$

(8) $$\begin{array}{ccc} {\hat{q}}_{1}\cdot {\hat{q}}_{2} & = & q_{1r}\cdot q_{2r}+\varepsilon \left(q_{1d}\cdot q_{2r}+q_{1r}\cdot q_{2d}\right)\in {\mathrm{DQ}}_{s} \end{array}$$

(9) $$\begin{array}{ccc} q_{1}\times q_{2} & = & \left(0,\xi_{1}{\bar{q}}_{2}+\xi_{2}{\bar{q}}_{1}+{\bar{q}}_{1}\times {\bar{q}}_{2}\right)\in H_{v} \end{array}$$

(10) $$\begin{array}{ccc} {\hat{q}}_{1}\times {\hat{q}}_{2} & = & q_{1r}\times q_{2r}+\varepsilon \left(q_{1d}\times q_{2r}+q_{1r}\times q_{2d}\right)\in {\mathrm{DQ}}_{v} \end{array}$$

The swap product of a dual quaternion is ${\hat{q}}^{s}=q_{d}+\varepsilon q_{r}\in \mathrm{DQ}$. The ⊙ product of a dual quaternion is $\hat{c}⊙\hat{q}=\left(c_{r}+\varepsilon c_{d}\right)⊙\left(q_{r}+\varepsilon q_{d}\right)=c_{r}q_{r}+\varepsilon c_{d}q_{d},\hat{q}\in \mathrm{DQ}$. The circle product of two dual quaternions is ${\hat{q}}_{1}\circ {\hat{q}}_{2}=q_{1r}\cdot q_{2r}+q_{1d}\cdot q_{2d},{\hat{q}}_{1},{\hat{q}}_{2}\in {\mathrm{DQ}}_{v}$.

The following properties can be shown, with the above definitions [28]:

(11) $$\hat{a}\circ \left(\hat{b}⊗\hat{c}\right)={\hat{b}}^{s}\circ \left({\hat{a}}^{s}⊗{\hat{c}}^{*}\right)={\hat{c}}^{s}\circ \left({\hat{b}}^{*}⊗{\hat{a}}^{s}\right)\in R,\;\hat{a},\hat{b},\hat{c}\in \mathrm{DQ}$$

(12) $$\hat{a}\circ \left(\hat{b}\times \hat{c}\right)={\hat{b}}^{s}\circ \left(\hat{c}\times {\hat{a}}^{s}\right)={\hat{c}}^{s}\circ \left({\hat{a}}^{s}\times \hat{b}\right),\;\hat{a},\hat{b},\hat{c}\in {\mathrm{DQ}}_{v}$$

(13) $${\hat{a}}^{s}\circ {\hat{b}}^{s}=\hat{a}\circ \hat{b},\;\hat{a},\hat{b}\in \mathrm{DQ}$$

(14) $$\left|\right|\hat{a}{\left|\right|}^{2}=\hat{a}\circ \hat{a},\;\hat{a}\in {\mathrm{DQ}}_{r}$$

A dual quaternion can be written as

(15) $$\hat{q}=q+\varepsilon \frac{1}{2}q⊗r^{i}$$

where q∈H is a unit quaternion representing rotation, and $r^{i}\in H_{v}$ is a vector quaternion representing translation. Then, the unit dual quaternion can represent transformation including both rotation and translation.

### 2.2. Graph Theory

Representing rigid bodies as nodes in a network topology graph, the information topology among them can be described by a graph. Let a undirected graph be defined by G(V,E), where V is a set of n∈N vertices (nodes) labeled as $\nu_{1},\nu_{2},\cdots ,\nu_{n}$, and $E\in \left(\nu_{i},\nu_{j}\right):\nu_{i},\nu_{j}\in V$ a finite set of edges. The adjacency matrix $A=\left[a_{ij}\right]\in R^{n\times n}$ is defined such that $a_{ij}=1$ if $(\nu_{i},\nu_{j})\in E$ and $a_{ij}=0$ otherwise.

### 2.3. Equations of Attitude-Orbit Coupled Relative Motion Based on Dual Quaternions

Let $F_{I}$ represent the Earth-centered inertial frame. The body-fixed coordinate system $F_{i}$ is solidly associated with the *i*th rigid body. Let $F_{di}$ represent the desired frame of *i*th rigid body.

The kinematics equation of the *i*-th rigid body based on dual quaternions can be described as [29]

(16) $${\dot{\hat{q}}}_{i}=\frac{1}{2}{\hat{q}}_{i}⊗{\hat{\omega }}_{i}^{i}$$

where ${\hat{q}}_{i}$ and ${\hat{\omega }}_{i}^{i}$ are the dual quaternion and the dual velocity of $F_{i}$ with respect to $F_{I}$, respectively. They are defined as

(17) $$\begin{array}{ccc} {\hat{q}}_{i} & = & q_{i}+\varepsilon \frac{1}{2}q_{i}⊗r_{i}^{i} \end{array}$$

(18) $$\begin{array}{ccc} {\hat{\omega }}_{i}^{i} & = & \omega_{i}^{i}+\varepsilon \left({\dot{r}}_{i}^{i}+\omega_{i}^{i}\times r_{i}^{i}\right) \end{array}$$

where $r_{i}^{i}$, ${\dot{r}}_{i}^{i}$ and $\omega_{i}^{i}$$\in H_{v}$ represent translation, linear velocity and angular velocity expressed in the $F_{i}$, respectively.

The dynamics of the *i*-th rigid body based on dual quaternions can be described as

(19) $${\hat{M}}_{i}{\dot{\hat{\omega }}}_{i}^{i}={\hat{F}}_{i}^{i}-{\hat{\omega }}_{i}^{i}\times {\hat{M}}_{i}{\hat{\omega }}_{i}^{i}$$

where ${\hat{F}}_{i}^{i}$ is the dual force acting on the *i*-th rigid body, ${\hat{M}}_{i}$ is the dual inertia matrix, which is defined as [30]

(20) $$\begin{array}{cc} {\hat{M}}_{i}\; & =m_{i}\frac{d}{d\varepsilon }I_{3}+\varepsilon J_{i}\\ \; & =\left[\begin{array}{ccc} m_{i}\frac{d}{d\varepsilon }+\varepsilon J_{i11} & \varepsilon J_{i12} & \varepsilon J_{i13}\\ \varepsilon J_{i21} & m_{i}\frac{d}{d\varepsilon }+\varepsilon J_{i22} & \varepsilon J_{i23}\\ \varepsilon J_{i31} & \varepsilon J_{i32} & m_{i}\frac{d}{d\varepsilon }+\varepsilon J_{i33} \end{array}\right] \end{array}$$

where $m_{i}$ and $J_{i}$ are the mass and inertia matrix of the *i*th rigid body, respectively. The inverse of ${\hat{M}}_{i}$ is defined as ${\hat{M}}_{i}^{-1}=J_{i}^{-1}\frac{d}{d\varepsilon }+\varepsilon \frac{1}{m_{i}}I_{3}$ [31].

In this paper, for the case of the space gravitational-wave detection system in Earth orbit, total dual forces acting on the spacecraft will be decomposed as follows

(21) $${\hat{F}}_{i}^{i}={\hat{f}}_{gi}^{i}+{\hat{f}}_{dsi}^{i}+{\hat{f}}_{J2i}^{i}+{\hat{f}}_{ui}^{i},\;i=1,2,3$$

where ${\hat{f}}_{gi}^{i}$, ${\hat{f}}_{dsi}^{i}$ and ${\hat{f}}_{J2i}^{i}$ represent the effect due to gravitational force, solar-pressure perturbation and $J_{2}$-perturbation force, respectively. Solar-irradiance fluctuations can be a significant disturbance for accurate orbit determination problems [32]. In the gravitational-wave detection project, the disturbance caused by solar-pressure perturbation cannot be ignored. ${\hat{f}}_{ui}^{i}=f_{ui}^{i}+\varepsilon \tau_{ui}^{i}$ represents the dual control force.

When the gravitational-wave mission is performed, the test mass will follow nearly free-fall trajectories. Here, we also do not consider the effect of non-conservative forces on the test mass. Hence, total dual forces acting on the test mass will be decomposed as follows

(22) $${\hat{F}}_{i}^{i}={\hat{f}}_{gi}^{i}+{\hat{f}}_{J2i}^{i}+{\hat{f}}_{ui}^{i},\;i=4,5,\cdots ,9$$

The dual forces ${\hat{f}}_{gi}^{i}$, ${\hat{f}}_{dsi}^{i}$ and ${\hat{f}}_{J2i}^{i}$ can be written as

(23) $$\begin{array}{ccc} {\hat{f}}_{gi}^{i} & = & m_{i}a_{gi}^{i}+\varepsilon \tau_{▽gi}^{i} \end{array}$$

(24) $$\begin{array}{ccc} {\hat{f}}_{dsi}^{i} & = & m_{i}a_{dsi}^{i}+\varepsilon 0 \end{array}$$

(25) $$\begin{array}{ccc} {\hat{f}}_{J2i}^{i} & = & m_{i}a_{J2i}^{i}+\varepsilon 0 \end{array}$$

where $a_{gi}^{i}$, $\tau_{▽gi}^{i}$, $a_{J2i}^{i}$ and $a_{dsi}^{i}$ are the gravitational acceleration, the gravity-gradient torque, the perturbing acceleration due to Earth’s oblateness, and the acceleration caused by solar radiation pressure, respectively, given by

(26) $$\begin{array}{ccc} a_{gi}^{i} & = & -\frac{\mu_{e}r_{i}^{i}}{∥r_{i}^{i}{∥}^{3}}-\mu_{m}\left(\frac{r_{i}^{i}-r_{m}^{i}}{∥r_{i}^{i}-r_{m}^{i}{∥}^{3}}+\frac{r_{m}^{i}}{∥r_{m}^{i}{∥}^{3}}\right)\\ \; & \; & -\mu_{s}\left(\frac{r_{i}^{i}-r_{s}^{i}}{∥r_{i}^{i}-r_{s}^{i}{∥}^{3}}+\frac{r_{s}^{i}}{∥r_{s}^{i}{∥}^{3}}\right) \end{array}$$

(27) $$\begin{array}{ccc} \tau_{▽gi}^{i} & = & 3\mu_{e}\frac{r_{i}^{i}\times J_{i}r_{i}^{i}}{∥r_{i}^{i}{∥}^{5}} \end{array}$$

(28) $$\begin{array}{ccc} a_{J2i}^{I} & = & -\frac{3}{2}\frac{\mu_{e}J_{2}R_{e}^{2}}{∥r_{i}^{I}{∥}^{5}}\left(D-5{\left(\frac{r_{i}^{z}}{r_{i}^{I}}\right)}^{2}I_{3}\right)r_{i}^{I} \end{array}$$

(29) $$\begin{array}{ccc} a_{dsi}^{i} & = & -P_{⊙}\frac{A}{m_{i}}\frac{r_{⊙}}{r_{⊙}^{3}}AU^{2}\left(1+\epsilon \right) \end{array}$$

where $\mu_{e}$ = 398,600.44190 ${\mathrm{km}}^{3}/s^{2}$ is Earth’s gravitational parameter, $\mu_{m}=4902.800076$${\mathrm{km}}^{3}/s^{2}$ is the Moon’s gravitational parameter, $\mu_{s}$ = 132,712,440,040.94400 ${\mathrm{km}}^{3}/s^{2}$ is the sun’s gravitational parameter; $r_{m}^{i}$ and $r_{s}^{i}$ denote the position vector of the Moon and sun, respectively. $R_{e}=6378.137$ km is the Earth’s mean equatorial radius, $J_{2}=0.0010826267$, D= diag{1,1,3}; $r_{i}^{I}={\left[r_{i}^{x},r_{i}^{y},r_{i}^{z}\right]}^{T}$ represents the coordinates of $r_{i}$ expressed in the inertial coordinate system. $P_{⊙}=4.56\times 10^{-6}$$N\cdot m^{2}$ is the solar radiation pressure at 1 AU (astronomical unit), *A* is the frontal area of the spacecraft, $r_{⊙}$ the position vector from the sun to the spacecraft, and ϵ the reflectivity of the surface.

By virtue of the dual quaternion algebra, the motion between the body-fixed frame and its desired frame can be expressed in the $F_{i}$ as the relative dual quaternion described by

(30) $${\hat{q}}_{ei}={\hat{q}}_{di}^{*}⊗{\hat{q}}_{i}=q_{ei}+\varepsilon \frac{1}{2}q_{ei}⊗r_{ei}^{i}$$

where ${\hat{q}}_{di}$ is the dual quaternion of $F_{di}$ with respect to $F_{I}$, and ${\hat{q}}_{di}^{*}$ is the conjugate of ${\hat{q}}_{di}$. $r_{ei}^{i}$ is the error position between the *i*-th rigid body and its desired position, given in $F_{i}$. $q_{ei}$ is the error quaternion of $F_{i}$ with respect to $F_{di}$. The relative kinematic and dynamic equations are given by

(31) $${\dot{\hat{q}}}_{ei}=\frac{1}{2}{\hat{q}}_{ei}⊗{\hat{\omega }}_{ei}^{i}$$

(32) $$\begin{array}{cc} {\hat{M}}_{i}{\dot{\hat{\omega }}}_{ei}^{i}= & {\hat{F}}_{i}^{i}-{\hat{\omega }}_{i}^{i}\times {\hat{M}}_{i}{\hat{\omega }}_{i}^{i}+{\hat{M}}_{i}\left({\hat{\omega }}_{ei}^{i}\times {\hat{\omega }}_{di}^{i}\right)\\ \; & -{\hat{M}}_{i}\left({\hat{q}}_{ei}^{*}⊗{\dot{\hat{\omega }}}_{di}^{di}⊗{\hat{q}}_{ei}\right) \end{array}$$

where ${\hat{\omega }}_{ei}^{i}$ is the dual velocity error between $F_{i}$ and $F_{di}$, expressed in $F_{i}$. ${\hat{\omega }}_{di}^{i}$ is the dual velocity of $F_{di}$ expressed in $F_{i}$, that is ${\hat{\omega }}_{di}^{i}={\hat{q}}_{ei}^{*}⊗{\hat{\omega }}_{di}^{di}⊗{\hat{q}}_{ei}$.

### 2.4. Problem Statement

**Figure 1:**
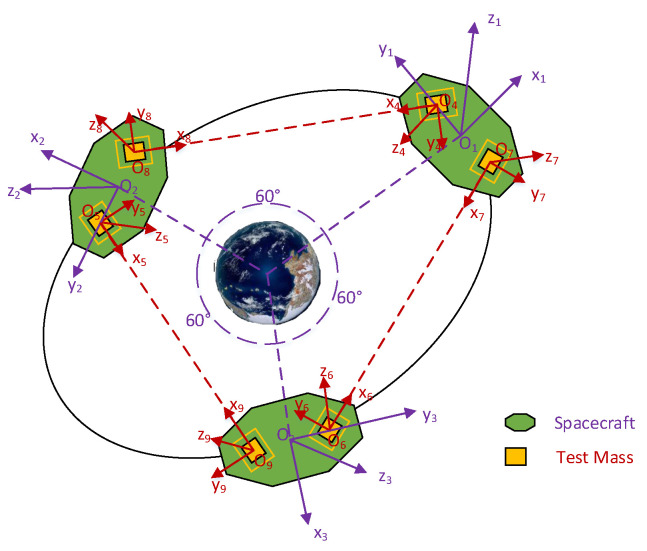
Desired formation configuration for the gravitational-wave detection system.

The space gravitational-wave detection project aims to verify general relativity. TianQin is a space-borne gravitational-wave detector in the millihertz frequencies, scheduled for launch in 2035 [4]. The project consists of three spacecrafts and six test masses, with an orbital radius of about 105 km. The distance between the spacecrafts will be monitored using laser interferometry. The formation configuration of the spacecrafts is required to be stable for the scientific mission [33]. Before a gravitational-wave detection mission can begin, the spacecraft needs to precisely enter a trajectory designed to meet the requirements of gravitational-wave detection. In this paper, the state that meets the requirements of the formation configuration is called the desired state, as shown in Figure 1. The test mass in the desired state is located at the center of the cavity. The science mission can only begin when both the spacecraft and the test mass are in the desired state. When there is a deviation between the actual state and the desired state, the coordinated control method is used to control the spacecraft and the test masses.

In the desired states, the relationships between the spacecrafts can be described as follows

(33) $$\begin{array}{cc} {\hat{q}}_{di+1} & ={\hat{q}}_{di}⊗{\hat{q}}_{sc},\;i=1,2\\ {\hat{q}}_{di-2} & ={\hat{q}}_{di}⊗{\hat{q}}_{sc},\;i=3 \end{array}$$

where ${\hat{q}}_{sc}=q_{sc}+\varepsilon \frac{1}{2}q_{sc}⊗r_{sc}$ denotes the dual quaternion of $F_{di}$ relative to $F_{di+1}$. $r_{sc}$ and $q_{sc}$ are the relative position vector and the quaternion of $F_{di}$ with respect to $F_{di+1}$, respectively. The relationships between the test masses and the spacecraft can be described by the following

(34) $$\begin{array}{cc} {\hat{q}}_{di+3} & ={\hat{q}}_{i}⊗{\hat{q}}_{tm1},\;i=1,2,3\\ {\hat{q}}_{di+6} & ={\hat{q}}_{i}⊗{\hat{q}}_{tm2},\;i=1,2,3 \end{array}$$

where ${\hat{q}}_{tm1}=q_{tm1}+\varepsilon \frac{1}{2}q_{tm1}⊗r_{tm1}$ and ${\hat{q}}_{tm2}=q_{tm2}+\varepsilon \frac{1}{2}q_{tm2}⊗r_{tm1}$ denote the dual quaternion of the spacecraft relative to the two test masses inside it, respectively. $r_{tm1}$ and $q_{tm1}$ are the relative position vector and the quaternion of $F_{i}$ with respect to $F_{di+3}$, respectively. $r_{tm2}$ and $q_{tm2}$ are the relative position vector and the quaternion of $F_{i}$ with respect to $F_{di+6}$, respectively.

The kinematics and dynamic models of the desired *i*-th rigid body in $F_{di}$ is similar to the *i*-th rigid body corresponding to Equations (16)–(19), where the notations ${\hat{q}}_{i}$, ${\hat{\omega }}_{i}^{i}$, $q_{i}$, $r_{i}^{i}$, $\omega_{i}^{i}$, and $F_{i}^{i}$ are replaced by ${\hat{q}}_{di}$, ${\hat{\omega }}_{di}^{di}$, $q_{di}$, $r_{di}^{di}$, $\omega_{di}^{di}$, and $F_{di}^{di}$, respectively. The total dual force applied to the desired *i*-th rigid body is independent of the dual control force, i.e., ${\hat{F}}_{di}^{di}={\hat{f}}_{gdi}^{di}+{\hat{f}}_{J_{2}di}^{di}$.

Consider the system given by Equations (31) and (32), use $\left({\hat{q}}_{di}\left(t\right),{\hat{\omega }}_{di}^{di}\left(t\right)\right)$ to denote the desired state of the *i*-th rigid body, and use $({\hat{q}}_{ei}\left(t\right)$, ${\hat{\omega }}_{ei}^{i}\left(t\right))$ to denote the relative motion error and velocity error, respectively. The objective of this paper is to design a distributed coordination control law ${\hat{f}}_{ui}^{i}$ based on dual quaternions such that the states of the rigid bodies $\left({\hat{q}}_{i}\left(t\right),{\hat{\omega }}_{i}^{i}\left(t\right)\right)$ can track their desired states $\left({\hat{q}}_{di}\left(t\right),{\hat{\omega }}_{di}^{di}\left(t\right)\right)$. In other words, the errors state $\left({\hat{q}}_{ei}\left(t\right),{\hat{\omega }}_{ei}^{i}\left(t\right)\right)$ of the closed-loop system are bounded and converge to an arbitrarily small neighborhood of the origin in the presence of communication delays. That is, when t→∞,

(35) $$\begin{array}{cc} {\hat{q}}_{ei}\left(t\right) & \to \pm \hat{1},\;i=1,2,...,9\\ {\hat{\omega }}_{ei}^{i}\left(t\right) & \to \hat{0},\;i=1,2,...,9 \end{array}$$

where $\hat{1}=1+\varepsilon 0\in \mathrm{DQ}$, $\hat{0}=0+\varepsilon 0\in \mathrm{DQ}$, $1=(1,\bar{0})\in H$ and $0=(0,\bar{0})\in H$, respectively.

## 3. Control Law Design

In this section, a gravitational-wave detection system with three rigid spacecrafts and six test masses tracking their desired reference state is considered. Our purpose is to design control schemes based on dual quaternion so that the spacecraft and test masses can converge to the desired state. Before moving on, the following assumptions and a lemma are provided.

**Assumption 1.**  *The spacecraft and test masses are regarded as rigid bodies, i = 1 ∼ 3 represents the spacecraft, and i = 4 ∼ 9 represents the test mass.*

**Assumption 2.**  *Each spacecraft and test mass can provide body-fixed control forces and control torques along three axes of its body frame.*

**Assumption 3.**  *The communication topology graph G is undirected and connected, and it does not change with time.*

**Assumption 4.**  *Full states of the rigid bodies are available.*

**Lemma 1**  ([34]). *The multi-agent system composed of n agents with system dynamics is given by*

(36) $$u_{i}=\xi_{i},\;i=1,\cdots ,n$$

*and a consensus algorithm is proposed as*

(37) $$u_{i}=-\sum_{j=1}^{n}a_{ij}\left(\xi_{i}-\xi_{j}\right)$$

*where $a_{ij}$ are the elements of the adjacency matrix A. Consensus is said to be reached among the n agents if $\xi_{i}\to \xi_{j}$, ∀i≠j.*

We extend the consistency algorithm of Lemma 1 to the rigid-body attitude-orbit coupled dynamic system. It is worth pointing out that the algorithm in Lemma 1 cannot be directly applied to rigid-body attitude-orbit coupling dynamic system due to the inherent nonlinear factors of attitude-orbit coupling dynamics. It is not obvious to extend the results of Lemma 1 to rigid-body attitude-orbit coupled dynamical systems. In addition, we consider that there is a constant communication delay between the spacecrafts. The coordinated control law is a feedback–feedforward strategy described by

(38) $$\begin{array}{cc} {\hat{f}}_{ui}^{i}= & -{\hat{k}}_{1i}⊙{\left({\hat{p}}_{ei}^{i}\right)}^{s}-{\hat{k}}_{2i}⊙{\left({\hat{\omega }}_{ei}^{i}\right)}^{s}+{\hat{\omega }}_{i}^{i}\times {\hat{M}}_{i}{\hat{\omega }}_{i}^{i}-\Gamma_{i}\\ \; & -{\hat{M}}_{i}\left({\hat{\omega }}_{ei}^{i}\times {\hat{\omega }}_{di}^{i}\right)+{\hat{M}}_{i}\left({\hat{q}}_{ei}^{*}⊗{\dot{\hat{\omega }}}_{di}^{di}⊗{\hat{q}}_{ei}\right)\\ \; & -{\hat{k}}_{3i}⊙\sum_{j=1}^{n}a_{ij}{\left({\hat{\omega }}_{ei}^{i}-{\hat{\omega }}_{ej}^{j}\left(t-T_{ij}\right)\right)}^{s} \end{array}$$

where ${\hat{k}}_{1i}=k_{1di}+\varepsilon k_{1ri}$, ${\hat{k}}_{2i}=k_{2di}+\varepsilon k_{2ri}$, ${\hat{k}}_{3i}=k_{3di}+\varepsilon k_{3ri}$ with $k_{1di}$, $k_{1ri}$, $k_{2di}$, $k_{2ri}$, $k_{3di}$, $k_{3ir}>0$. $T_{ij}$ is the communication delay from the *j*-th to *i*-th rigid body. ${\hat{p}}_{ei}^{i}$ is defined as

(39) $$\begin{array}{c} {\hat{p}}_{ei}^{i}={\bar{p}}_{ei}+\varepsilon \frac{1}{2}r_{ei}^{i} \end{array}$$

where ${\bar{p}}_{ei}$ is the vector part of the quaternion $q_{ei}$.

$\Gamma_{i}$ denotes total dual force other than dual control force. For i=1∼3, $\Gamma_{i}$ represents the dual force acting on the spacecraft,

(40) $$\begin{array}{cc} \Gamma_{i}=\; & {\hat{f}}_{gi}^{i}+{\hat{f}}_{dsi}^{i}+{\hat{f}}_{J2i}^{i} \end{array}$$

otherwise, for i=4∼9, $\Gamma_{i}$ represents the dual force acting on the test mass,

(41) $$\begin{array}{cc} \Gamma_{i}= & {\hat{f}}_{gi}^{i}+{\hat{f}}_{J2i}^{i} \end{array}$$

**Assumption 5.**  *It is assumed that there is a constant communication delay T>0 between neighbor spacecrafts, and there is no communication delay between a spacecraft and test mass. Therefore, $T_{ij}=T$ with i,j=1∼3,i≠j. Otherwise, $T_{ij}=0$.*

Note that negative feedback of $-{\hat{k}}_{1i}⊙{\left({\hat{p}}_{ei}^{i}\right)}^{s}-{\hat{k}}_{2i}⊙{\left({\hat{\omega }}_{ei}^{i}\right)}^{s}$ is the absolute position and attitude tracking item and is used to track the overall desired position and attitude of the multi rigid-body system. $-{\hat{k}}_{3i}⊙\sum_{j=1}^{n}a_{ij}{\left({\hat{\omega }}_{ei}^{i}-{\hat{\omega }}_{ej}^{j}\left(t-T_{ij}\right)\right)}^{s}$ is the relative position and attitude keeping to ensure that the relative position and attitude of the multi rigid-body remain consistent. The remaining terms involved in Equation (38) are used to compensate for the dual force induced by Earth’s gravitational force and torque, $J_{2}$-perturbation force, Moon’s gravitational force, Sun’s gravitational force and solar pressure perturbation. The stability of the resultant closed-loop system is stated in the following theorem.

**Theorem 1.**  *Consider a closed-loop system described by Equations*(31), (32) and (38)*. If Assumptions*A1–A5 *are valid, the states ${\hat{q}}_{ei}$ and ${\hat{\omega }}_{ei}^{i}$ are uniformly bounded and consensus tracking is asymptotically achieved, that is, ${\hat{q}}_{ei}\to \pm \hat{1}$ and ${\hat{\omega }}_{ei}^{i}\to \hat{0}$ as t→∞.*

**Proof**  (Proof of Theorem 1). Consider a Lyapunov function candidate as follows

(42) $$\begin{array}{ccc} V_{1} & = & \sum_{i=1}^{n}{\hat{k}}_{1i}⊙\left({\hat{q}}_{ei}-\hat{1}\right)\circ \left({\hat{q}}_{ei}-\hat{1}\right)+\frac{1}{2}\sum_{i=1}^{n}{\left({\hat{\omega }}_{ei}^{i}\right)}^{s}\circ \left({\hat{M}}_{i}{\hat{\omega }}_{ei}^{i}\right)\\ & & +\frac{1}{2}\sum_{i=1}^{n}\sum_{j=1}^{n}a_{ij}{\hat{k}}_{3i}⊙{\left(\int_{t-T_{ij}}^{t}{\hat{\omega }}_{ej}^{j}\left(\tau \right)\circ {\hat{\omega }}_{ej}^{j}\left(\tau \right)d\tau \right)}^{s} \end{array}$$

which satisfies $V_{1}\geq 0$ and $V_{1}=0$ if and only if $\left({\hat{q}}_{ei},{\hat{\omega }}_{ei}^{i}\right)\left(t\right)=\left(\hat{1},\hat{0}\right)$.

Differentiating $V_{1}$ with respect to time, we can obtain

(43) $$\begin{array}{ccc} {\dot{V}}_{1} & = & \sum_{i=1}^{n}{\hat{k}}_{1i}⊙\left({\dot{\hat{q}}}_{ei}\circ \left({\hat{q}}_{ei}-\hat{1}\right)+\left({\hat{q}}_{ei}-\hat{1}\right)\circ {\dot{\hat{q}}}_{ei}\right)+\sum_{i=1}^{n}{\left({\hat{\omega }}_{ei}^{i}\right)}^{s}\circ {\hat{M}}_{i}{\dot{\hat{\omega }}}_{ei}^{i}\\ & & +\frac{1}{2}\sum_{i=1}^{n}\sum_{j=1}^{n}a_{ij}{\hat{k}}_{3i}⊙\left({\hat{\omega }}_{ej}^{j}\circ {\hat{\omega }}_{ej}^{j}-{\hat{\omega }}_{ej}^{j}\left(t-T_{ij}\right)\circ {\hat{\omega }}_{ej}^{j}\left(t-T_{ij}\right)\right)\\ & = & 2\sum_{i=1}^{n}{\hat{k}}_{1i}⊙\left({\hat{q}}_{ei}-\hat{1}\right)\circ {\dot{\hat{q}}}_{ei}+\sum_{i=1}^{n}{\left({\hat{\omega }}_{ei}^{i}\right)}^{s}\circ {\hat{M}}_{i}{\dot{\hat{\omega }}}_{ei}^{i}\\ & & +\frac{1}{2}\sum_{i=1}^{n}\sum_{j=1}^{n}a_{ij}{\hat{k}}_{3i}⊙\left({\hat{\omega }}_{ej}^{j}\circ {\hat{\omega }}_{ej}^{j}-{\hat{\omega }}_{ej}^{j}\left(t-T_{ij}\right)\circ {\hat{\omega }}_{ej}^{j}\left(t-T_{ij}\right)\right)\\ & = & \sum_{i=1}^{n}{\hat{k}}_{1i}⊙\left({\hat{q}}_{ei}-\hat{1}\right)\circ \left({\hat{q}}_{ei}⊗{\hat{\omega }}_{ei}^{i}\right)+\sum_{i=1}^{n}{\left({\hat{\omega }}_{ei}^{i}\right)}^{s}\circ {\hat{M}}_{i}{\dot{\hat{\omega }}}_{ei}^{i}\\ & & +\frac{1}{2}\sum_{i=1}^{n}\sum_{j=1}^{n}a_{ij}{\hat{k}}_{3i}⊙\left({\hat{\omega }}_{ej}^{j}\circ {\hat{\omega }}_{ej}^{j}-{\hat{\omega }}_{ej}^{j}\left(t-T_{ij}\right)\circ {\hat{\omega }}_{ej}^{j}\left(t-T_{ij}\right)\right) \end{array}$$

Applying Equation (11), the first item in Equation (43) yields

(44) $$\begin{array}{ccc} & & \sum_{i=1}^{n}{\hat{k}}_{1i}⊙\left({\hat{q}}_{ei}-\hat{1}\right)\circ \left({\hat{q}}_{ei}⊗{\hat{\omega }}_{ei}^{i}\right)\\ & = & \sum_{i=1}^{n}{\hat{k}}_{1i}⊙{\left({\hat{\omega }}_{ei}^{i}\right)}^{s}\circ \left({\hat{q}}_{ei}^{*}⊗{\left({\hat{q}}_{ei}-\hat{1}\right)}^{s}\right)\\ & = & \sum_{i=1}^{n}{\left({\hat{\omega }}_{ei}^{i}\right)}^{s}\circ {\hat{k}}_{1i}⊙{\left({\hat{p}}_{ei}^{i}\right)}^{s} \end{array}$$

The second item in Equation (43) yields

(45) $$\begin{array}{ccc} \sum_{i=1}^{n}{\left({\hat{\omega }}_{ei}^{i}\right)}^{s}\circ {\hat{M}}_{i}{\dot{\hat{\omega }}}_{ei}^{i} & = & \sum_{i=1}^{n}{\left({\hat{\omega }}_{ei}^{i}\right)}^{s}\circ (-{\hat{k}}_{1i}⊙{\left({\hat{p}}_{ei}^{i}\right)}^{s}-{\hat{k}}_{2i}⊙{\left({\hat{\omega }}_{ei}^{i}\right)}^{s}\\ & & -{\hat{k}}_{3i}⊙\sum_{j=1}^{n}a_{ij}{\left({\hat{\omega }}_{ei}^{i}-{\hat{\omega }}_{ej}^{j}\left(t-T_{ij}\right)\right)}^{s}) \end{array}$$

Then, substituting Equations (44) and (45) into Equation (43), we have

(46) $$\begin{array}{ccc} {\dot{V}}_{1} & = & -\sum_{i=1}^{n}{\left({\hat{\omega }}_{ei}^{i}\right)}^{s}\circ \left({\hat{k}}_{2i}⊙{\left({\hat{\omega }}_{ei}^{i}\right)}^{s}\right)\\ & & -\sum_{i=1}^{n}{\left({\hat{\omega }}_{ei}^{i}\right)}^{s}\circ {\hat{k}}_{3i}⊙\sum_{j=1}^{n}a_{ij}{\left({\hat{\omega }}_{ei}^{i}-{\hat{\omega }}_{ej}^{j}\left(t-T_{ij}\right)\right)}^{s}\\ & & +\frac{1}{2}\sum_{i=1}^{n}\sum_{j=1}^{n}a_{ij}{\hat{k}}_{3i}⊙\left({\hat{\omega }}_{ej}^{j}\circ {\hat{\omega }}_{ej}^{j}-{\hat{\omega }}_{ej}^{j}\left(t-T_{ij}\right)\circ {\hat{\omega }}_{ej}^{j}\left(t-T_{ij}\right)\right) \end{array}$$

Note that the undirected topology is balanced, meaning that $\sum_{j=1}^{n}a_{ij}=\sum_{j=1}^{n}a_{ji}$ for i=1,⋯,n; then, it follows that

(47) $$\begin{array}{c} \sum_{i=1}^{n}\sum_{j=1}^{n}a_{ij}{\hat{\omega }}_{ei}^{i}=\sum_{j=1}^{n}\sum_{i=1}^{n}a_{ji}{\hat{\omega }}_{ei}^{i}=\sum_{i=1}^{n}\sum_{j=1}^{n}a_{ij}{\hat{\omega }}_{ej}^{j} \end{array}$$

Applying Equation (13), Equation (46) yields

(48) $$\begin{array}{ccc} {\dot{V}}_{1} & = & -\sum_{i=1}^{n}{\left({\hat{\omega }}_{ei}^{i}\right)}^{s}\circ \left({\hat{k}}_{2i}⊙{\left({\hat{\omega }}_{ei}^{i}\right)}^{s}\right)-\frac{1}{2}\sum_{i=1}^{n}\sum_{j=1}^{n}a_{ij}{\hat{k}}_{3i}⊙\left({\hat{\omega }}_{ei}^{i}\circ {\hat{\omega }}_{ei}^{i}\right)\\ & & -\frac{1}{2}\sum_{i=1}^{n}\sum_{j=1}^{n}a_{ij}{\hat{k}}_{3i}⊙\left({\hat{\omega }}_{ej}^{j}\left(t-T_{ij}\right)\circ {\hat{\omega }}_{ej}^{j}\left(t-T_{ij}\right)\right)\\ & & +\sum_{i=1}^{n}\sum_{j=1}^{n}a_{ij}{\hat{k}}_{3i}⊙\left({\hat{\omega }}_{ei}^{i}\circ {\hat{\omega }}_{ej}^{j}\left(t-T_{ij}\right)\right)\\ & = & -\frac{1}{2}\sum_{i=1}^{n}\sum_{j=1}^{n}a_{ij}{\hat{k}}_{3i}⊙\left({\hat{\omega }}_{ei}^{i}-{\hat{\omega }}_{ej}^{j}\left(t-T_{ij}\right)\right)\circ \left({\hat{\omega }}_{ei}^{i}-{\hat{\omega }}_{ej}^{j}\left(t-T_{ij}\right)\right)\\ & & -\sum_{i=1}^{n}{\hat{k}}_{2i}⊙\left({\hat{\omega }}_{ei}^{i}\circ {\hat{\omega }}_{ei}^{i}\right)\leq 0 \end{array}$$

Therefore, $\underset{t\to \infty }{lim}V_{1}\left(t\right)$ exists and is finite. The states ${\hat{q}}_{ei}$ and ${\hat{\omega }}_{ei}^{i}$ are uniformly bounded. In addition, the boundedness of ${\hat{q}}_{ei}$ and ${\hat{\omega }}_{ei}^{i}$ means that ${\dot{\hat{\omega }}}_{ei}^{i}$ and ${\dot{\hat{q}}}_{ei}$ are bounded. Hence, by Barbalat’s lemma, ${\hat{q}}_{ei}\to \pm \hat{1}$ and ${\hat{\omega }}_{ei}^{i}\to \hat{0}$ as t→∞. We complete the proof. □

**Remark 1.**  *According to Ref. [28], both of the equilibrium points ${\hat{q}}_{ei}=\hat{1}$ and ${\hat{q}}_{ei}=-\hat{1}$ represent the same relative position and attitude between frames, and they are acceptable. However, this can lead to an unwinding phenomenon where large angles are performed before coming to $\hat{1}$ under the proposed algorithm. The solutions to this problem are given by Refs. [35,36], and we omit discussion of methods to deal with the problem.*

## 4. Numerical Simulations

In this section, the proposed controller is applied to the Earth-centered orbital space gravitational-wave detection system, which involves three spacecrafts and six test masses and tracking their respective desired attitudes and positions. The initial conditions are assumed to be as follows [37]: the three spacecrafts are isomorphic, the masses are all 650 kg, and the inertia matrix is $J_{i}$ (i=1∼3); The six test masses are isomorphic, the masses are all 2.45 kg, and the inertia matrix is $J_{i}$ (i=4∼9).

(49) $$\begin{array}{cc} J_{i} & =\left[\begin{array}{ccc} 162.5 & 3 & 2\\ 3 & 162.5 & 2.5\\ 2 & 2.5 & 325\; \end{array}\right]\mathrm{kg}\cdot m^{2},\;i=1∼3\\ J_{i} & =\left[\begin{array}{ccc} 0.001 & 0 & 0\\ 0 & 0.001 & 0\\ 0 & 0 & 0.001\; \end{array}\right]\mathrm{kg}\cdot m^{2},\;i=4∼9 \end{array}$$

**Figure 2:**
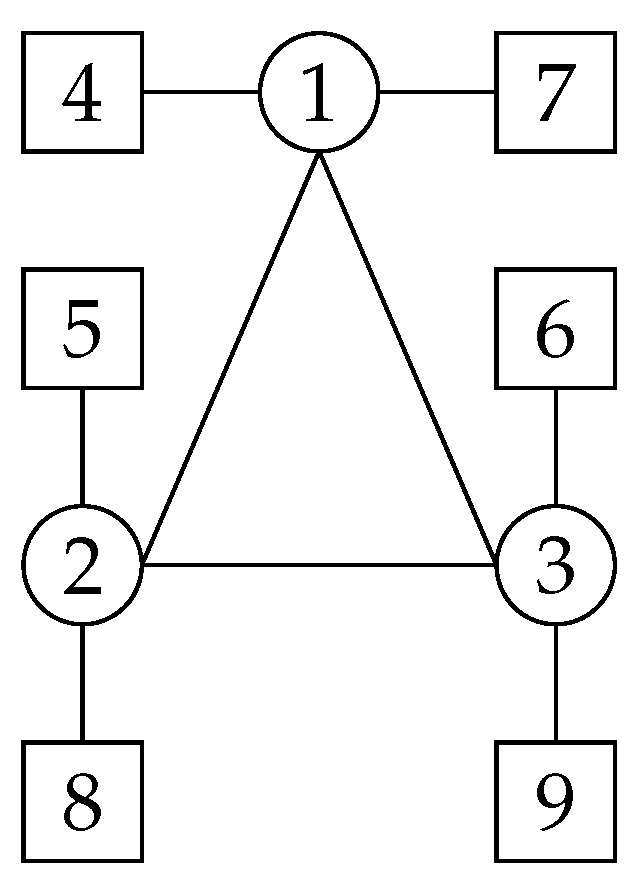
Information topology of the spacecraft formation system.

The information topology G in the spacecraft formation system is shown in Figure 2. Serial numbers 1, 2, and 3 represent spacecrafts SC1 ∼ 3, and the remaining serial numbers represent test masses TM1 ∼ 6. The desired orbit of the spacecrafts is shown in Table 1. The initial position errors, velocity errors, angular velocity errors, and quaternion errors are presented in Table 2. $q_{sc}=\left[\cos\left(30\right),0,0,\sin\left(30\right)\right]$, $r_{sc}={\left[0,1.73118,0\right]}^{T}\times 10^{8}$ m, $q_{tm1}=\left[\cos\left(75\right),0,0,\sin\left(75\right)\right]$, $q_{tm2}=\left[\cos\left(105\right),0,0,\sin\left(105\right)\right]$. The relative attitude and position are measurable and assumed to be normally distributed. The attitude and position standard errors of the spacecraft are 1 μrad and 1 m, respectively. The attitude and position standard errors of test mass are 200 nrad and 1.7 nm, respectively [38]. The communication delay T=0.67 s. In this paper, simulations are validated using thrusts of 100 μN and 100 mN, respectively. Here, 100 μN corresponds to the case of formation station-keeping, where a micro propulsion system is used to compensate for the non-conservative forces in the system during the space gravitational-wave detection mission. Here, 100 mN corresponds to the case of formation reconfiguration. A greater thrust is needed to ensure that the spacecrafts enter the scientific-mission stage.

### 4.1. The Maximum Available Control Force Is 100 μN

In this subsection, the maximum available control force and torque of spacecraft are assumed to be $f_{sc\_max}$ = 100 μN and $\tau_{sc\_max}$ = 100 μN·m, the maximum available control force and torque of test masses are assumed to be $f_{tm\_max}$ = 0.7 μN and $\tau_{tm\_max}$ = 0.7 μN·m, respectively. Thus, $\left|\right|f_{ui}^{i}\left|\right|\leq f_{sc\_max}$ and $\left|\right|\tau_{ui}^{i}\left|\right|\leq \tau_{sc\_max}\left(i=1∼3\right)$, $\left|\right|f_{ui}^{i}\left|\right|\leq f_{tm\_max}$ and $\left|\right|\tau_{ui}^{i}\left|\right|\leq \tau_{tm\_max}\left(i=4∼9\right)$. Using a trial-and-error procedure, the gains for the controller (38) are selected as $k_{1di}=10^{-6}$, $k_{1ri}=7\times 10^{-5}$, $k_{2di}=5\times 10^{-2}$, $k_{2ri}=6\times 10^{-2}$, $k_{3di}=6\times 10^{-6}$, $k_{3ri}=10^{-7}$, i=1∼3. $k_{1di}=10^{-3}$, $k_{1ri}=7\times 10^{-5}$, $k_{2di}=5\times 10^{-2}$, $k_{2ri}=6\times 10^{-3}$, $k_{3di}=6\times 10^{-6}$, $k_{3ri}=10^{-9}$, i=4∼9.

**Figure 3:**
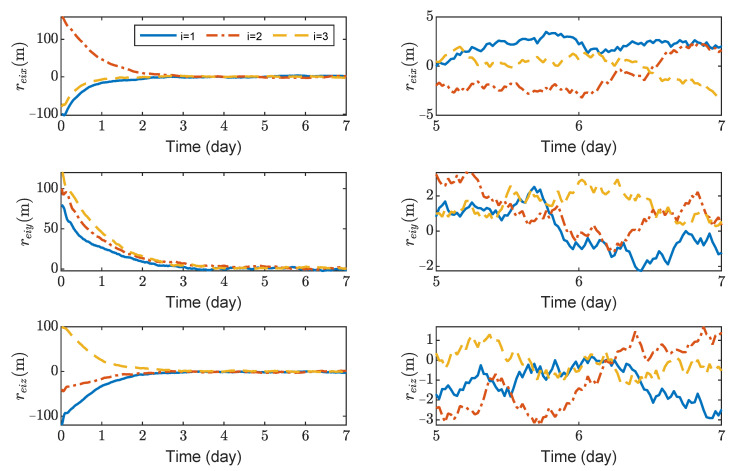
Relative position errors of spacecraft SC1∼3, the max control force of spacecraft is 100 μN.

**Figure 4:**
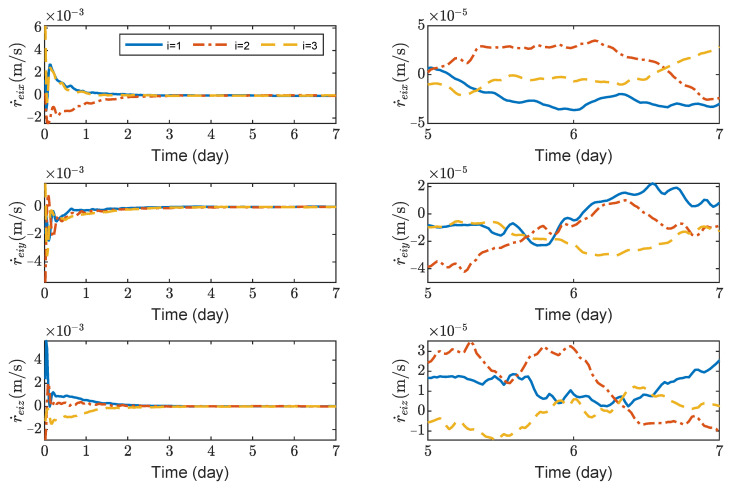
Relative linear velocity errors of spacecraft SC1∼3, the max control force of spacecraft is 100 μN.

**Figure 5:**
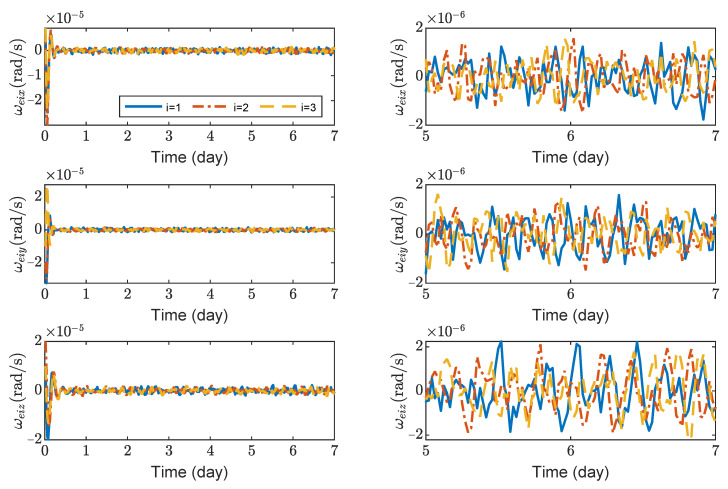
Relative angular velocity errors of spacecraft SC1∼3, the max control torque of spacecraft is 100 μN· m.

**Figure 6:**
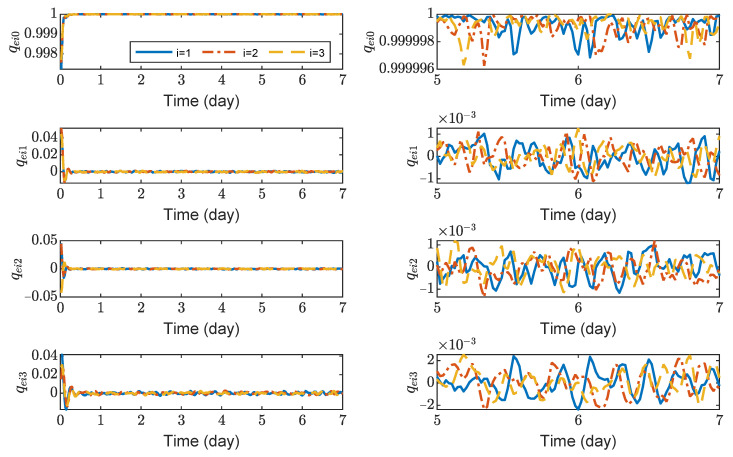
Relative attitude errors of spacecraft SC1 ∼ 3, the max control torque of spacecraft is 100 μN· m.

Figure 3, Figure 4, Figure 5 and Figure 6 show the relative position errors, relative velocity errors, relative angular velocity errors, and relative attitude errors of the three spacecrafts, respectively. It can be seen that the spacecrafts can asymptotically track their desired positions and attitudes, and the tracking errors can converge to the region $|r_{eiw}|<5$ m, $|{\dot{r}}_{eiw}|<5\times 10^{-5}$ m/s, $|\omega_{eiw}|<2\times 10^{-6}$ rad/s, (w=x,y,z), and $|q_{eik}|<2\times 10^{-3},\left(k=1,2,3\right)$. The control accuracy of position and attitude is of the same order of magnitude as the measurement error. The transient phase and final accuracy of position tracking and attitude tracking are acceptable. It can be seen from Figure 3, Figure 4, Figure 5 and Figure 6 that the relative attitude error and the relative angular velocity error converge faster than the relative position error, and the convergence time for translation and rotation is about 3 days and 1 day, respectively.

**Figure 7:**
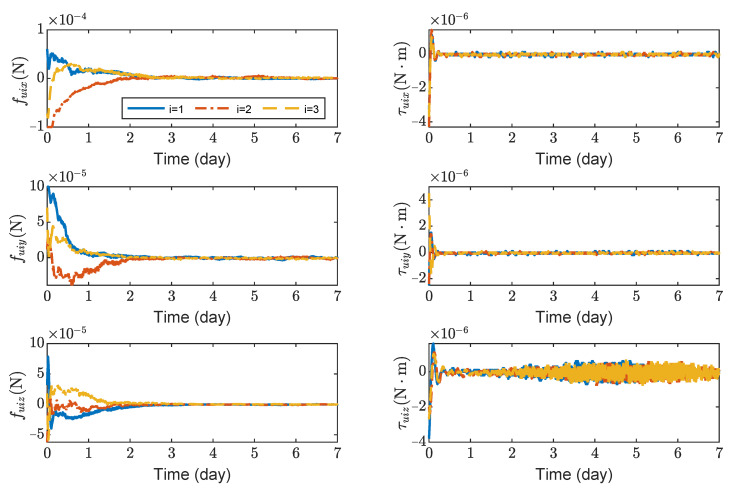
Control forces and control torques about SC1 ∼ 3, the max control force of spacecraft is 100 μN.

Figure 7 shows the variation curves of control forces and control torques of spacecrafts SC1 ∼ 3. It can be seen that the maximum control forces of the spacecrafts are $10^{-4}$ N, and the maximum control torques are $5\times 10^{-6}$N·m without saturation. As seen in Figure 7, the steady-state error of $\tau_{uiz}$ is significantly larger than $\tau_{uix}$ and $\tau_{uiy}$. This is because $J_{33}$ is larger than $J_{11}$ and $J_{22}$ in the inertia matrix $J_{i}$ (i=1∼3).

**Figure 8:**
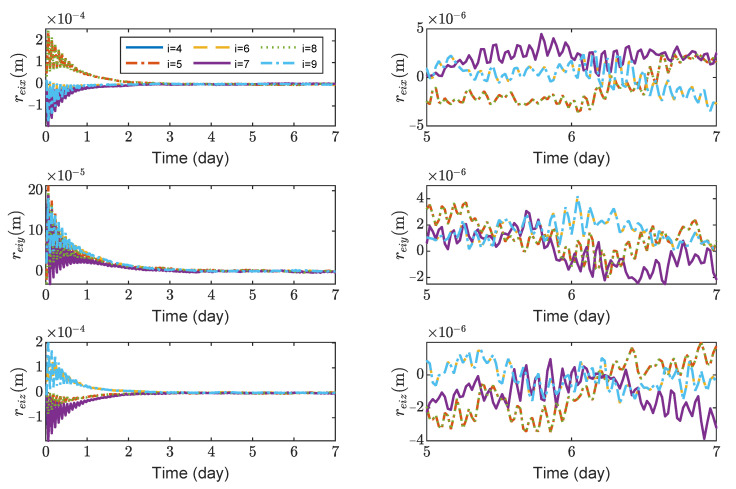
Relative position errors of TM1 ∼ 6, the max control force of test mass is 0.2 μN.

**Figure 9:**
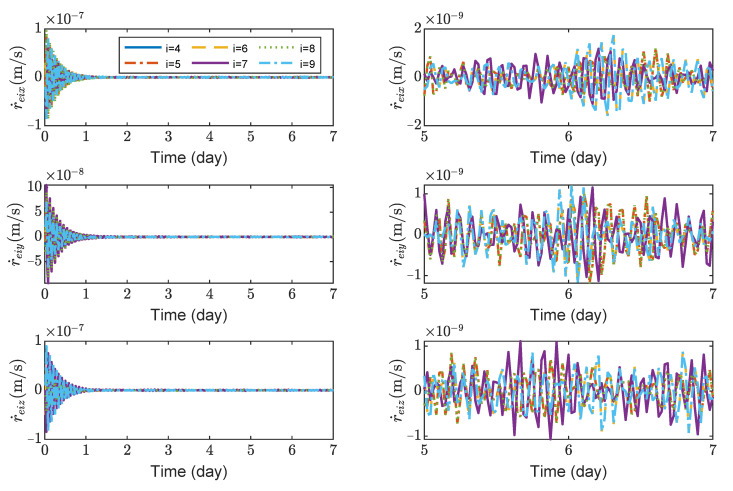
Relative linear velocity errors of spacecrafts TM1 ∼ 6, the max control force of spacecraft is 100 μN.

**Figure 10:**
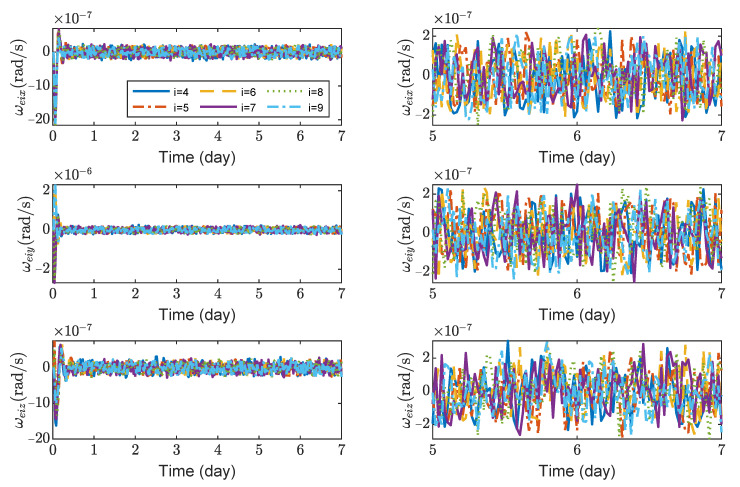
Relative angular velocity errors of spacecrafts TM1 ∼ 6, the max control torque of spacecraft is 100 μN· m.

**Figure 11:**
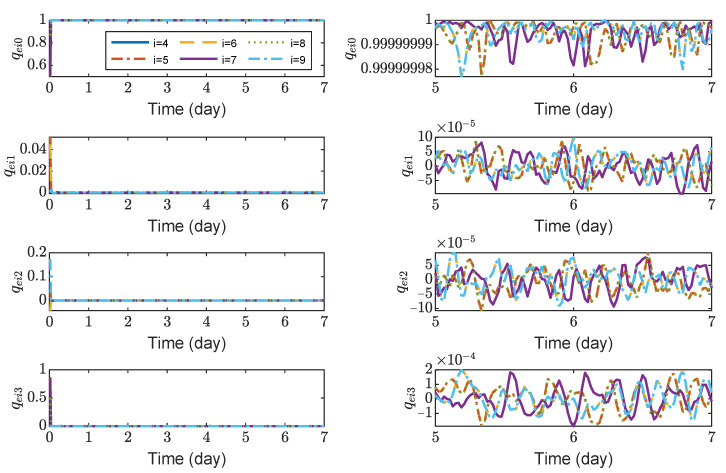
Relative attitude errors of spacecrafts TM1 ∼ 6, the max control torque of spacecraft is 100 μN· m.

Figure 8, Figure 9, Figure 10 and Figure 11 show the relative position errors, relative velocity errors, relative angular velocity errors and relative attitude errors of the six test masses, respectively. It can be seen that the test masses can asymptotically track their desired positions and attitudes, and the tracking errors can converge to the region $|r_{eiw}|<5\times 10^{-6}$ m, $|{\dot{r}}_{eiw}|<2\times 10^{-9}$ m/s, $|\omega_{eiw}|<3\times 10^{-7}$ rad/s, (w=x,y,z), $|q_{eik}|<2\times 10^{-4}$, (k=1,2,3). The maximum range of test-mass position tracking errors are 250, 250 and 250 μm, respectively. In order to prevent collisions of the test masses when tracking the spacecrafts, the minimum size of the cavity in which the test mass is located should be [500 + *L*, 500 + *L*, 500 + *L*] μm (*L* is the side length of the test mass). It can be seen from Figure 8 and Figure 9 that in the steady-state stage, the position errors and velocity errors of the two test masses in the same spacecraft tend to be the same. This indicates that the test masses achieve the tracking of the spacecraft.

**Figure 12:**
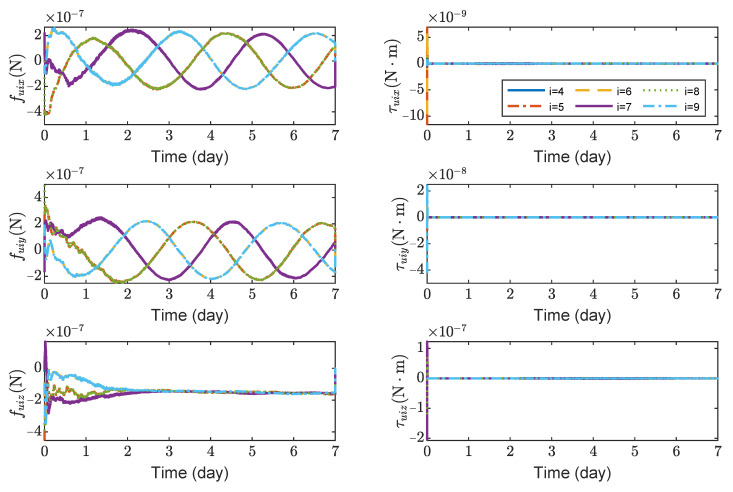
Control forces and control torques of TM1 ∼ 6, the max control force of spacecraft is 100 μN.

Figure 12 shows the variation curves of control forces and control torques of test masses TM1 ∼ 6. It can be seen that the maximum control forces of the test masses are $5\times 10^{-7}$ N and the maximum control torques are $2\times 10^{-7}$N·m without saturation. Figure 12 indicates that the effect of solar pressure needs to be compensated when test masses are tracking spacecrafts. Note that this paper only focuses on the control of the spacecraft and the test masses before the start of the detection mission. After starting the detection mission, the test masses are in a drag-free state.

This method achieves consistent tracking control of spacecrafts and test masses when the maximum actuator output of the spacecraft is 100 μN. The settling time for the spacecraft and the test masses in translational motion is about 4 days. However, for rotational motion, tge spacecraft’s settling time is about 1 day and the test masses’ settling time is about 3 days. This is because the actuators of the test masses are electrostatic actuators, making it take longer to track the spacecraft’s attitude.

### 4.2. The Maximum Available Control Force Is 100 mN

In order to complete the formation reconfiguration as soon as possible, we assume that the spacecraft can provide a larger control force and control torque. In this subsection, the maximum available control forces and torques of spacecraft are assumed to be $f_{sc\_max}$ = 100 mN and $\tau_{sc\_max}$ = 100 mN·m, respectively. Thus, $\left|\right|f_{ui}^{i}\left|\right|\leq f_{sc\_max}$ and $\left|\right|\tau_{ui}^{i}\left|\right|\leq \tau_{sc\_max}\left(i=1∼3\right)$. Using a trial-and-error procedure, the gains for the controller (38) are selected as $k_{1di}=0.03$, $k_{1ri}=0.035$, $k_{2di}=5$, $k_{2ri}=6$, $k_{3di}=0.01$, $k_{3ri}=0.006$, and i=1∼3. In order to prevent the test mass from colliding with the cavity, the Cage and Vent Mechanism [39] was used to fix the test mass. Hence, only three spacecraft simulations are presented in this subsection.

**Figure 13:**
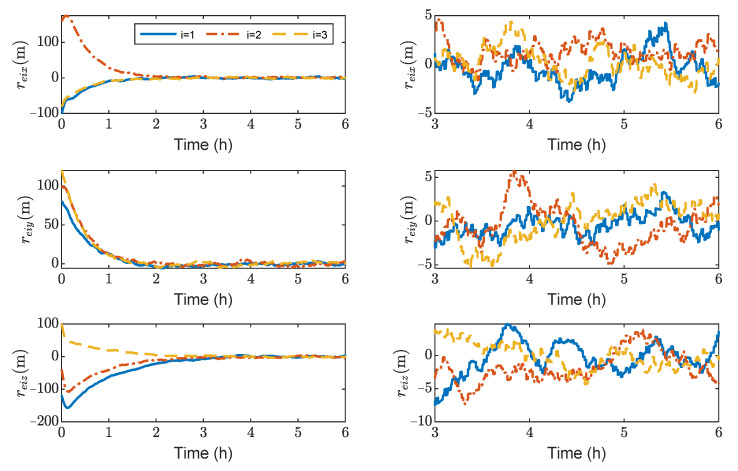
Relative position errors of spacecraft SC1∼3, the max control force of spacecraft is 100 mN.

**Figure 14:**
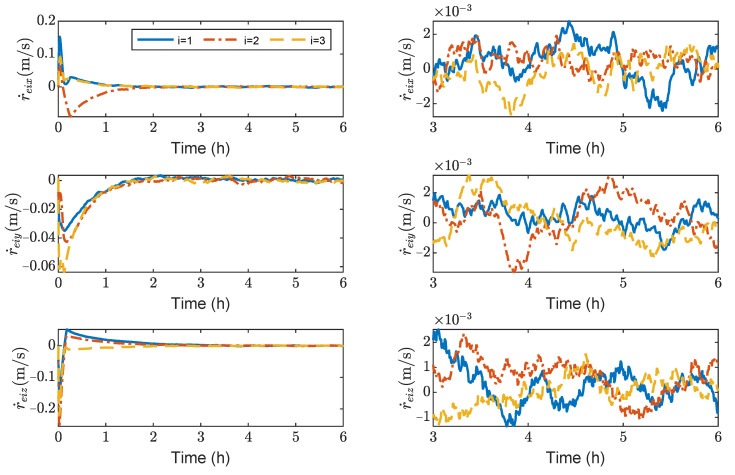
Relative linear velocity errors of spacecraft SC1∼3, the max control force of spacecraft is 100 mN.

**Figure 15:**
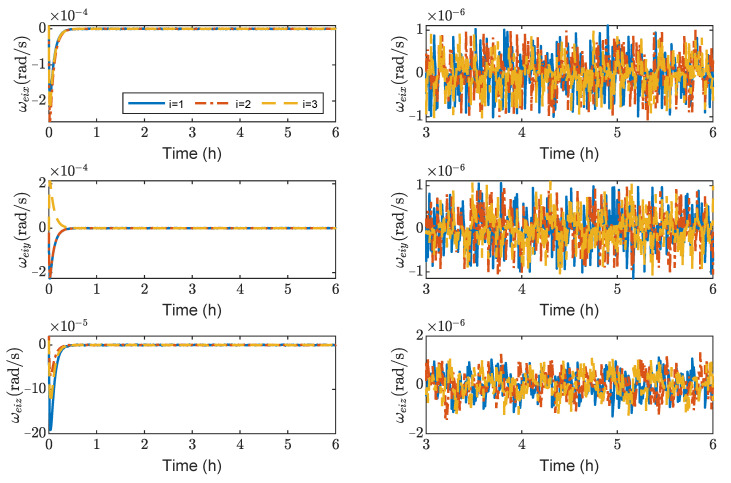
Relative angular velocity errors of spacecraft SC1∼3, the max control torque of spacecraft is 100 mN· m.

**Figure 16:**
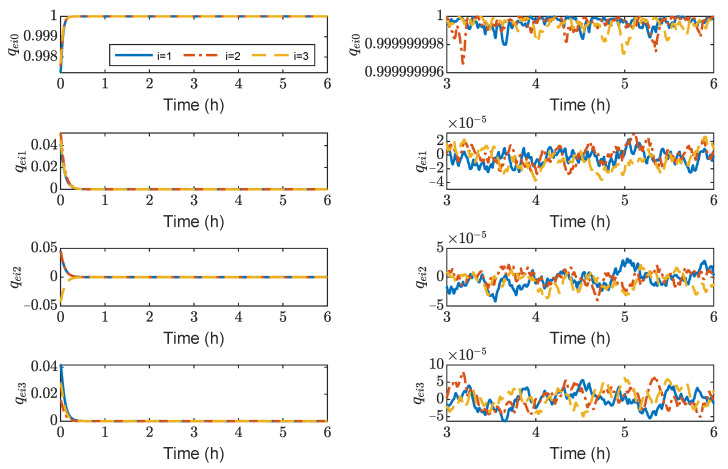
Relative attitude errors of spacecraft SC1 ∼ 3, the max control torque of spacecraft is 100 mN· m.

**Figure 17:**
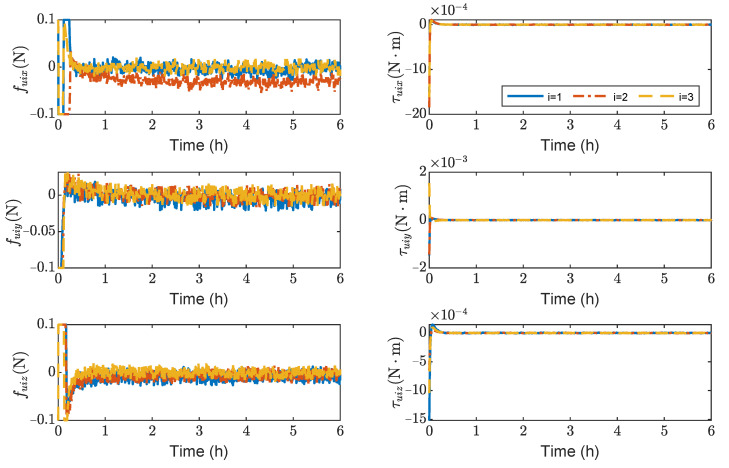
Control forces and control torques about SC1 ∼ 3, the max control force of spacecraft is 100 mN.

Figure 13, Figure 14, Figure 15 and Figure 16 show the time histories of the position errors, velocity errors, angular velocity errors and relative attitude errors of each spacecraft with communication delays, respectively. It can be seen that the spacecraft can asymptotically track their desired positions and desired attitudes, and the tracking errors can converge to the region $|r_{eiw}|<6$ m, $|{\dot{r}}_{eiw}|<3\times 10^{-3}$ m/s,$|\omega_{eiw}|<2\times 10^{-6}$ rad/s, (w=x,y,z), $|q_{eik}|<5\times 10^{-5},\left(k=1,2,3\right)$. The control forces and control torques of the spacecrafts can be seen in Figure 17, which indicates that the control forces and control torques can stay within the limitation of 100 mN and 1 mN·m, respectively.

As shown in Figure 13, Figure 14, Figure 15 and Figure 16, it can be seen that the convergence times are about 3 h and 1 h for the translation and rotation, respectively. It saves at least 20 times the time compared to using the microthruster system. However, the control accuracy of position and attitude errors is lower than that of the microthruster system. When using actuators with a larger thrust, the test masses need to be fixed. However, frequently fixing and releasing test masses will affect their accuracy.

## 5. Conclusions

A distributed coordination control law based on a dual-quaternion description model is proposed for a gravitational-wave detection formation system in this paper. The novelty of this paper lies in the combination of the full-state feedback controller and the consistency algorithm to design a unified form of attitude orbit coupling coordination controller. Furthermore, the asymptotic stability of the closed-loop system is guaranteed, and the coordinated control of the desired position and attitude of the spacecrafts and the test masses is achieved considering communication delays.

The following conclusions can be drawn from the simulation results:

(1) The spacecraft can control the position and attitude of the spacecraft and the test masses simultaneously using the microthruster during the maneuver, but it takes at least 3 days under the initial error of about 100 m;
(2) Increasing the thrust shortens the control time, but the test masses need to be fixed to prevent the test masses from colliding with the cavity during the orbit transfer.

The above conclusions can provide a blueprint for the development of a control strategy for the spacecrafts in gravitational-wave detection missions: As an important part of the inertial sensor, the test masses are frequently locked, which will reduce their accuracy. Therefore, when the spacecraft has an orbit entry error, a larger thrust can be used for precise orbit and attitude corrections to achieve a certain accuracy before releasing the test masses and then using the electrostatic force provided by the capacitive sensors to control the test masses. Once the test masses are released, the microthruster system can be used to correct the attitude and orbit of the spacecraft to the desired state. Future developments will consider the model uncertainties of the spacecraft and the time-vary communication delays between the spacecrafts.

## Figures and Tables

**Table 1:** Desired orbital parameters.

Parameter	Value	Unit
Perigee altitude	$9.999\times 10^{7}$	m
Eccentricity	0.00043	-
Inclination	74.5362	
Argument of perigee	346.5528	
RAAN	211.6003	
True anomaly (SC1)	61.3296	
True anomaly (SC2)	181.3296	
True anomaly (SC3)	301.3296	

**Table 2:** Initial conditions.

	Initial Position Error (m)	Initial Velocity Error ($m\cdot s^{-1}$)	Initial Angular Velocity Error ($\mathrm{Rad}\cdot s^{-1}$)	Initial Quaternion Error (−)
1(SC1)	[−100 80 −120]${}^{T}$	[3 −2 1]${}^{T}\times 10^{-3}$	[0.8 −2 1] ${}^{T}\times 10^{-5}$	[0.9972 0.0416 0.0454 0.0416]
2(SC2)	[160 100 −40]${}^{T}$	[−1 −2 1] ${}^{T}\times 10^{-3}$	[0.7 −2 2] ${}^{T}\times 10^{-5}$	[0.9976 0.0515 0.0445 0.0151]
3(SC3)	[−80 120 100]${}^{T}$	[4 0 −1] ${}^{T}\times 10^{-3}$	[0.9 −1 1] ${}^{T}\times 10^{-5}$	[0.9977 0.0447 −0.0424 0.0280]
4(TM1)	[−3 2 −2]${}^{T}\times 10^{-5}$	[3 −2 −1]${}^{T}\times 10^{-8}$	[0.8 −2 1]${}^{T}\times 10^{-9}$	[0.9972 0.0416 0.0454 0.0416]
5(TM2)	[5 −2 1]${}^{T}\times 10^{-5}$	[−2 −1 -1]${}^{T}\times 10^{-8}$	[1 −3 2]${}^{T}\times 10^{-9}$	[0.9976 0.0515 0.0445 0.0151]
6(TM3)	[2 7 5]${}^{T}\times 10^{-5}$	[3 1 −5]${}^{T}\times 10^{-8}$	[3 −1 5]${}^{T}\times 10^{-9}$	[0.9977 0.0447 −0.0424 0.0280]
7(TM4)	[−5 6 −2]${}^{T}\times 10^{-5}$	[−5 6 0]${}^{T}\times 10^{-8}$	[0.8 −3 4]${}^{T}\times 10^{-9}$	[0.5000 0 0 0.8660]
8(TM5)	[−2 −3 −1]${}^{T}\times 10^{-5}$	[−2 −3 −1]${}^{T}\times 10^{-8}$	[2 −5 3]${}^{T}\times 10^{-9}$	[0.8660 0 0 0.5000]
9(TM6)	[−2 2 1]${}^{T}\times 10^{-5}$	[−2 2 1]${}^{T}\times 10^{-8}$	[0.7 −6 8]${}^{T}\times 10^{-9}$	[0.9848 0 0.1736 0]

## Data Availability

Not applicable.

## Nomenclature

A	adjacency matrix (with entries $\left[a_{ij}\right]$)
$a_{gi}^{i}$	gravitational acceleration expressed in the body-fixed frame
$a_{J2i}^{i}$	perturbing acceleration due to Earth’s oblateness expressed in the body-fixed frame
$a_{dsi}^{i}$	the acceleration caused by solar radiation pressure expressed in the body-fixed frame
DQ, ${\mathrm{DQ}}_{v}$	set of dual quaternions and dual vector quaternions, respectively
${\mathrm{DQ}}_{s}$, ${\mathrm{DQ}}_{r}$	set of dual scalar quaternions and dual scalar quaternions with zero dual part, respectively
E	set of edges
$F_{I}$	the Earth-centered inertial frame
$F_{i}$, $F_{di}$	the body-fixed frame and the desired body-fixed frame of the *i*th rigid body, respectively
${\hat{F}}_{i}^{i}$, ${\hat{F}}_{di}^{di}$	total dual force expressed in the frames $F_{i}$ and $F_{di}$, respectively
${\hat{f}}_{ui}^{i}$	dual control force expressed in the body-fixed frame
${\hat{f}}_{gi}^{i}$, ${\hat{f}}_{gdi}^{di}$	dual gravitational force expressed in the frame $F_{i}$ and $F_{di}$, respectively
${\hat{f}}_{dsi}^{i}$	dual solar pressure perturbation expressed in the body-fixed frame
${\hat{f}}_{J2i}^{i}$, ${\hat{f}}_{J2di}^{di}$	dual $J_{2}$-perturbation force expressed in the frame $F_{i}$ and $F_{di}$, respectively
H, $H_{v}$, $H_{s}$	set of quaternions, set of vector quaternions, set of scalar quaternions
$I_{3}$	the 3-by-3 identity matrix
$J_{i}$, $m_{i}$	inertia matrix and mass of *i*-th rigid body
${\hat{k}}_{1i},{\hat{k}}_{2i},{\hat{k}}_{3i}$	control gains
${\hat{M}}_{i}$	dual inertia matrix
$q_{di}$, $q_{i}$	quaternion of the frames $F_{di}$ and $F_{i}$ with respect to the frame $F_{I}$
${\hat{q}}_{i}$	dual quaternion of the frame $F_{i}$ with respect to the frame $F_{I}$
$q_{i}^{*}$, ${\hat{q}}_{i}^{*}$	the conjugate of $q_{i}$ and ${\hat{q}}_{i}$
${\hat{q}}_{ei}$	dual quaternion of the frame $F_{i}$ with respect to the frame $F_{di}$
$T_{ij}$	the communication delay between the *j*-th rigid body and the *i*-th rigid body
$\omega_{i}^{i}$	angular velocity of $F_{i}$ frame with respect to the $F_{I}$ frame expressed in the $F_{i}$ frame
${\hat{\omega }}_{i}^{i}$	dual velocity of the frame $F_{i}$ with respect to the $F_{I}$ frame expressed in the $F_{i}$ frame
${\hat{\omega }}_{ei}^{i}$	dual velocity of the frame $F_{i}$ with respect to the frame $F_{di}$ frame expressed in the $F_{i}$ frame
$\mu_{e}$	Earth’s gravitational parameter
$\mu_{m}$	Moon’s gravitational parameter
$\mu_{s}$	sun’s gravitational parameter
V	set of vertices
ε	dual unit
